# Surfactin from *Bacillus subtilis* 1–4 disrupts the cell membrane of zoonotic S*taphylococcus intermedius in vitro* and maintains gut microbiota community in urban pigeons

**DOI:** 10.1186/s13099-026-00830-8

**Published:** 2026-04-01

**Authors:** Jia-Ho Shiu, Bo-Chen Huang, Hui-Jye Chen, Yo-Chia Chen

**Affiliations:** 1 General Research Service Center, National Pingtung University of Science and Technology, Pingtung, 91201 Taiwan (R.O.C.); 2 Department of Biological Science and Technology, National Pingtung University of Science and Technology, Pingtung, 91201 Taiwan (R.O.C.); 3 Graduate Institute of Biomedical Sciences, China Medical University, Taichung, 40402 Taiwan

**Keywords:** *Bacillus subtilis*, Surfactin, *Staphylococcus*, Gut microbiota stability

## Abstract

**Background:**

Urban pigeons frequently carry zoonotic, antibiotic-resistant *Staphylococcus* spp. and are increasingly recognized as reservoirs of multidrug-resistant (MDR) gut pathogens with One Health implications. Probiotic *Bacillus* strains and their lipopeptide products, such as surfactin, are promising antibiotic-sparing alternatives, but their in vivo effects on host gut microbiota and pathogen carriage in apparently healthy birds remain poorly characterized.

**Results:**

To explore alternative antimicrobial strategies, *Bacillus subtilis* 1–4 was isolated from soil and shown to produce surfactin, a potent antimicrobial peptide. In vitro, surfactin exhibited bactericidal activity against *S. aureus* and *S. intermedius* by compromising membrane integrity. To evaluate its in vivo effects, pigeons (*Columba livia*) were treated with surfactin for seven days, and changes in their gut microbiota were characterized using full-length 16 S rRNA gene sequencing. While microbial α- and β-diversity remained largely unchanged, the surfactin-treated microbiota displayed enhanced compositional stability relative to controls.

**Conclusions:**

These findings support surfactin’s potential as a targeted antimicrobial agent with minimal disruption to the host gut microbiota.

**Supplementary Information:**

The online version contains supplementary material available at 10.1186/s13099-026-00830-8.

## Introduction

Pigeons (*Columba livia*) are a widespread bird species in urban regions, which frequently share environments with humans and serve as potential reservoirs of bacteria carrying resistance and virulence factor genes [1]. These birds not only contribute to zoonotic infections but also pose significant public health concerns due to their role in the transmission and persistence of drug-resistant pathogens in shared ecosystems [2]. Previous studies have isolated methicillin-resistant *Staphylococcus intermedius* from pigeons since 2007 [3] and 3 different multidrug-resistant pathogens as well: *Staphylococcus aureus*,* Escherichia coli*, and *Candida albicans* [1]. These findings underscore the importance of developing alternative, antibiotic-sparing antimicrobial strategies for managing drug-resistant pathogens in pigeons and limiting their contribution to zoonotic transmission.

*S. intermedius* is a zoonotic pathogen known to cause infections in humans, birds, and mammals [4]. It has been identified as a primary cause of human wound or mucosal infections following animal contact [5, 6]. Moreover, co-infections involving *S. aureus* and *S. intermedius* have been reported [7], with their differing antibiotic sensitivities complicating treatment. Excessive use of antibiotics in these cases further exacerbates the problem by promoting the transfer of resistance genes between pathogens.

The inherent resilience of *Bacillus* spp., along with their ability to modulate gut microbiota and inhibit pathogenic colonization, underscores their utility in both biomedical and agricultural contexts [8, 9]. In modern poultry and livestock production, *Bacillus*-based direct-fed microbials—particularly *B. subtilis*—are widely incorporated into commercial diets as prophylactic alternatives to antibiotic growth promoters [10–12]. Numerous in vivo studies in broilers and pigs show that dietary *B. subtilis* supplementation improves growth performance, feed efficiency, and intestinal integrity while reshaping the gut microbiota, typically enriching lactic acid– and short-chain–fatty-acid–producing taxa and reducing Enterobacteriaceae and other potential pathogens [13–15], thereby improving colonization resistance against gut pathogens. However, many of these findings come from studies in which animals were experimentally infected with, or naturally exposed to, enteric pathogens (e.g., *Eimeria* spp., *Clostridium perfringens*, *Salmonella* spp.), and systematic evaluations of how prophylactic *Bacillus* supplementation influences the gut microbiota of apparently healthy hosts in the absence of defined pathogenic infection remain relatively limited and sometimes inconsistent [11, 15].

Antimicrobial peptides (AMPs), particularly those produced by *Bacillus subtilis*, such as surfactin, have garnered significant attention as potential alternatives to conventional antibiotics due to their targeted antibacterial activity and reduced propensity for inducing resistance [16, 17]. Surfactin, a cyclic lipopeptide originally isolated from *B. subtilis*, exhibits potent antimicrobial effects against a broad spectrum of pathogens [17–19] and possesses remarkable physicochemical stability and structural diversity, rendering it an attractive candidate for biotechnological and pharmaceutical applications [20]. However, the molecular mechanisms underlying its antibacterial activity—particularly against *S. intermedius*—are not yet fully elucidated, warranting further investigation. Furthermore, surfactin is commonly synthesized by diverse *Bacillus* species and is also noted for its probiotic potential in animal nutrition [21]. Despite the extensive in vitro characterization of surfactin, its safety profile, therapeutic efficacy, and influence on host-associated gut microbiota remain insufficiently explored in vivo.

In this study, we aimed to isolate and identify a new strain of *B. subtilis* from soil, characterize its production of surfactin, and examine its antibacterial activity against *S. intermedius*. The antimicrobial peptides produced by these strains were identified as surfactin, and their mechanisms of antimicrobial activity were investigated. We also investigated the effects of surfactin on the gut microbial communities of pigeons. Understanding these effects could provide valuable insights into the potential use of surfactin as a safe and effective antimicrobial agent. These findings may inform future strategies for mitigating the risks of antibiotic resistance in shared human-animal ecosystems.

## Materials and methods

### Screening and identification of bacterial isolates with antimicrobial activity

Soil samples were collected from the campus of National Pingtung University of Science and Technology (Pingtung, Taiwan), suspended in sterile water, serially diluted, and plated on Luria-Bertani agar (LA; 10 g/L tryptone, 5 g/L yeast extract, 5 g/L NaCl, 15 g/L agar). Colonies were isolated on LA and screened for antagonism using an indicator-agar (seeded-agar pour) assay against *Staphylococcus aureus* and *Staphylococcus intermedius* (BCRC 12154 and 12157; FIRDI, Hsinchu, Taiwan). Indicator strains were grown in LB at 37 °C (225 rpm) for 16 h, then 500 µL culture was mixed with 25 mL molten LB agar maintained at 55 °C to prepare uniform lawns. Candidate isolates were transferred by lightly spot-inoculating a single colony with a sterile loop, incubated at 37 °C for 16–18 h, and inhibition zones were recorded. Sixteen isolates with inhibition zones were re-screened, and isolate 1–4 consistently showed the strongest inhibition (Supplementary Fig. S1) and was selected for downstream identification.

Morphological characteristics of isolate 1–4 were examined from fresh colonies grown on LA, including colony appearance and cell morphology under light microscopy after Gram staining [22] Endospore formation was assessed using the Schaeffer–Fulton endospore stain (malachite green with heat as the primary stain and safranin as the counterstain) [23]. The biochemical/carbohydrate utilization profile was determined using API^®^ 50 CHB strips (bioMérieux) following the manufacturer’s instructions.

Genomic DNA from isolate 1–4 was extracted using the Tissue & Cell Genomic DNA Purification Kit (GMbiolab, Taiwan). The 16 S rDNA region was amplified using universal primers V1F (5′-AGAGTTTGATCCTGGCTCAG-3′) and V8R (5′-GACGGGGCGGTGWGRTC-3′). PCR products measuring 1365 bp were purified (BioKit Bio-C300), cloned into the yT&A vector (Yeastern Biotech, Taiwan), and transformed into *E. coli* DH5α. Recombinant plasmids were extracted (BioKit Bio-P300), and inserts were sequenced via Sanger sequencing (Genomics BioSci & Tech, Taiwan). Phylogenetic analysis of isolate 1–4 was conducted by aligning its 16 S rDNA sequence with 14 reference *Bacillus* spp. sequences from GenBank. A neighbor-joining tree was constructed using the Tamura–Nei model with 1,000 bootstrap replicates [24].

### Purification and analysis of antimicrobial peptides

AMPs were extracted from the bacterial isolate following cultivation in 300 mL of LB broth at 28 °C with agitation at 160 rpm for 48 h. The culture was centrifuged at 10,000 rpm for 30 min to remove cellular debris, and the supernatant was collected. The supernatant was subsequently acidified to pH 2 using formic acid and incubated at 4 °C overnight to facilitate peptide precipitation. The resulting pellet was recovered by centrifugation (10,000 rpm, 30 min, 4 °C) and dried using a rotary evaporator at 40 °C and 900 rpm. The dried pellet was then resuspended in methanol. The methanolic extract was passed through a 0.22 μm pore size filter and stored at 4 °C prior to further analysis [25]. The antibacterial activity of AMPs was assessed using a disk diffusion assay [26]. Overnight cultures of *S. aureus* and *S. intermedius* were prepared, and 0.1 mL of each was uniformly spread onto LA plates. Sterile paper discs (8 mm diameter; Advantec, Toyo Roshi Kaisha Ltd., Tokyo, Japan) were impregnated with AMPs dissolved in methanol at concentrations of 3.75, 7.5, and 15 µg/mL. An identical loading volume (50 µL per disc) was used for all treatments, positive controls, and negative controls (methanol alone). Following incubation at 37 °C for 16 h, zones of inhibition surrounding the discs were observed and measured to evaluate antibacterial efficacy.

The analysis of AMPs was performed by a Chromaster^®^ HPCL (Hitachi Co, Tokyo, Japan) equipped with a C18 column (250 × 4.6 mm, 5 μm, Mightysil) and a UV detector. The surfactin standard purchased from Sigma-Aldrich (S3523) was used as previously described [27]. For HPLC analysis, the methanolic AMP extract was diluted 10-fold before injection, and an identical injection volume of 10 µL was used for all runs. For comparison, surfactin standards were prepared at 2–10 mg/mL, and a 10 mg/mL surfactin standard was used for chromatographic (retention-time/peak) comparison. The size of AMPs was estimated using Tricine-sodium dodecyl sulfate–polyacrylamide gel electrophoresis (Tricine-SDS-PAGE) with Coomassie Blue staining (Supplementary Fig. S2).

### Determination of minimum inhibitory concentration (MIC) and minimum bactericidal concentration (MBC).

The MIC was defined as the lowest concentration of AMPs that prevented visible bacterial growth. Antibacterial activity was assessed against *S. aureus* and *S. intermedius* using a broth microdilution assay in 96-well plates. Overnight cultures of each strain were diluted in LB broth to a final concentration of 10⁶ CFU/mL. AMPs were added to each well to achieve final concentrations ranging from 0 to 0.8 mg/mL, with a total volume of 200 µL per well. Plates were incubated at 37 °C, and bacterial growth was monitored by measuring optical density at 600 nm at 12 h.

The minimum bactericidal concentration (MBC) was defined as the lowest concentration of AMPs that resulted in ≥ 99% bacterial mortality. To determine the MBC, aliquots (100 µL) from wells without visible growth were serially diluted 100- and 1,000-fold, and 100 µL of each dilution was spread onto LB agar plates. Following incubation at 37 °C for 24 h, colony counts were used to calculate bacterial survival rates and identify the MBC.

### Bacterial membrane-disruptive activity against *Staphylococcus* spp.

*S. intermedius* and *S. aureus* cells (OD₆₀₀ = 0.05) suspended in 100 µL TBS buffer (6.05 g/L Tris base, 0.8.76 g/L sodium chloride, pH 7.6) were dispensed into 96-well plates along with 5 µM Sytox Green reagent (S7020, Thermo Fisher Scientific, Waltham, MA, USA). Plates were incubated in the dark at room temperature for 15 min. Subsequently, 100 µL of AMPs were added to reach their respective MBCs. Fluorescence was measured spectrophotometrically (excitation: 480 nm; emission: 522 nm) at 30-minute intervals for 60 min, and again at 240 min in a microplate reader (Varioskan™ LUX, Vantaa, Finland) [28].

*S. intermedius* and *S. aureus* cells (OD₆₀₀ = 0.05) were suspended in 2 mL of TBS supplemented with AMPs at their respective MBC. The same volume of methanol without AMP was applied to the blank group. The suspensions were incubated at 37 °C with agitation at 225 rpm for 1 h. Following incubation, samples were centrifuged at 5000 rpm for 10 min at 4 °C, and the resulting supernatants were collected. Proteins released into the supernatants were subsequently analyzed by SDS–PAGE. For the blank control, AMPs were replaced with an equal volume of methanol [29].

Protein bands of interest were excised from the acrylamide gel using a sterile scalpel and transferred to sterile microcentrifuge tubes. In-gel protein digestion and subsequent mass spectrometry analysis were conducted by the Instrument Center of National Pingtung University of Science and Technology (Pingtung, Taiwan). Peptide analysis was performed using an electrospray ionization quadrupole time-of-flight (ESI-Q-TOF) mass spectrometer (ABI QSTAR Pulsar i System, Applied Biosystems, CA, USA) coupled with an UltiMate Capillary LC System (Thermo Finnigan, CA, USA). Mass spectra of trypsin-digested peptides from *S. intermedius* and *S. aureus* were analyzed against the NCBInr database using the Mascot search algorithm for protein identification [30].

### The intestinal bacterial communities of surfactin-fed pigeons

Animal protocols were approved by the IACUC of National Pingtung University of Science and Technology (Protocol No. NPUST-110-087). Our method is also reported in accordance with ARRIVE guidelines (https://arriveguidelines.org/). Eight healthy 6-month-old pigeons (*Columba livia*) purchased from Linluo pigeon farm (Pingtung, Taiwan) were randomly assigned to control and surfactin-treated groups (*n* = 4 per group). Birds were housed separately under ambient conditions to avoid cross-contamination. The experimental group (D0S and D7S) received surfactin (1 g/kg, w/w) via feed; the control group (D0CT and D7CT) received the basal diet alone, comprising maize (29%), wheat (19%), peas (37%), red sorghum (9%), white sorghum (4%), and safflower seeds (2%). Individual body weights were monitored during the 7-day feeding period, with identification maintained via color bands. Fecal samples were collected pre- (day 0; D0S and D0CT group) and post-treatment (day 7; D7S and D7CT group), then stored at − 20 °C. Sample handling and downstream microbiota analyses were blinded to minimize bias.

Genomic DNA was extracted using the QIAamp DNA Stool Mini Kit (QIAGEN, Germany). Near full-length 16 S rRNA genes were amplified using primers 27 F and 1492R, prepared with the SMRTbell Express Template Prep Kit 2.0 (Pacific Biosciences), purified (AMPure PB beads), and sequenced on the Sequel II platform (Biotools, Taiwan). Raw PacBio reads were filtered (RQ ≥ 30; ~1,500 bp), denoised, and chimera-checked using DADA2 (v1.20). Amplicon sequence variants (ASVs) were imported into QIIME2 (v2021.4; https://qiime2.org/) for downstream analysis. Taxonomic classification was conducted using the SILVA database (v138). Alpha diversity (e.g., Shannon index) was assessed, with significance evaluated via Kruskal–Wallis and Wilcoxon signed-rank tests (*p* < 0.05). Beta diversity was analyzed using weighted UniFrac distances [31] and visualized via Principal Coordinates Analysis (PCoA). Group differences in community structure were tested using ANOSIM. A species-level heatmap of the top 35 most abundant taxa was constructed. Abundances were square root-transformed, z-score normalized, and clustered using Bray–Curtis similarity (samples) and Pearson correlation (species) with the pheatmap package in R [32, 33]. The Linear Discriminant Analysis Effect Size (LEfSe) analysis (LDA > 4.0; *p* < 0.05) identified differentially abundant taxa among groups [34].

### Statistical analysis

All experiments were carried out in triplicate, and the data were presented as means ± standard deviation (SD). Data were analyzed by analysis of variance (ANOVA) using GraphPad Prism 5 (GraphPad Software LLC, San Diego, CA, USA) and were evaluated statistically significant at *p* < 0.05 by Tukey’s post hoc test.

## Results

### *Bacillus subtilis* isolate 1–4 with antibacterial activity

**Fig. 1:**
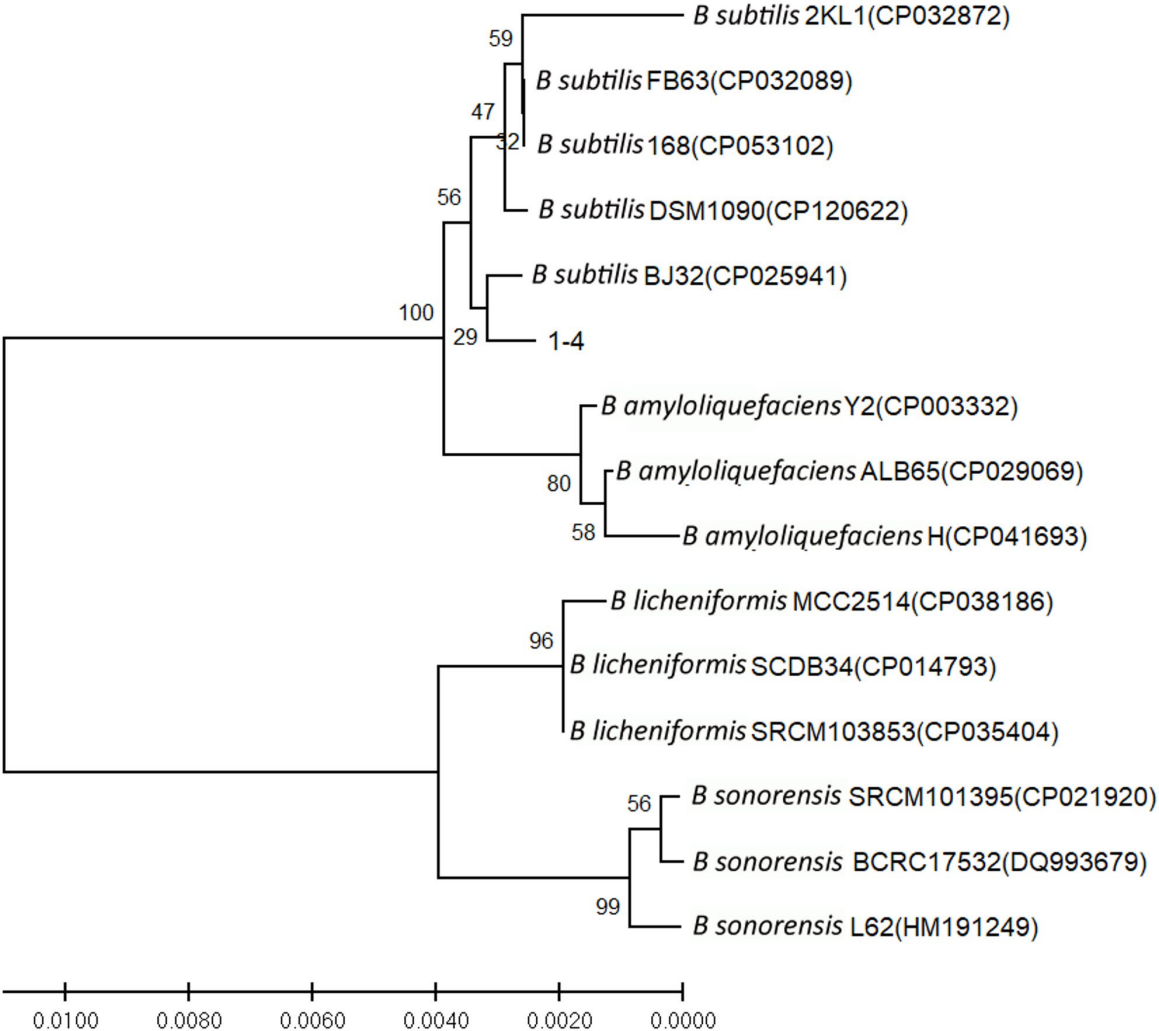
Phylogenetic tree of isolate 1–4 and related *Bacillus* species constructed based on the full length of 16 S rRNA gene sequences. The tree was generated using the neighbor-joining method with 1,000 bootstrap replicates, and bootstrap values > 50% are shown at the nodes. The GenBank accession numbers of reference sequences are indicated in parentheses. Scale bar represents the number of substitutions per nucleotide position

Isolate 1–4 was obtained from a soil sample cultivated on LA plates, and its antibacterial activity against *S. aureus* and *S. intermedius* was examined. Phylogenetic analysis based on 16 S rDNA sequences was conducted using the Neighbor-Joining algorithm with reference sequences retrieved from NCBI (Fig. 1). Fourteen bacilli, encompassing *B. subtilis*, *B. amyloliquefaciens*, *B. licheniformis*, and *B. sonorensis*, were grouped into four distinct clades. Isolate 1–4 was clustered within the *B. subtilis* clade (bootstrap support = 56%). Notably, the bootstrap support for the internal node linking isolate 1–4 as sister to the reference strain BJ32 was low (29), and thus the 16 S rDNA tree provides weak support for this specific closest-neighbor relationship within the B. subtilis complex. The morphological and biochemical characteristics of isolate 1–4—including Gram-positive rod shape, endospore formation, and carbon source utilization patterns—were also consistent with those of *B. subtilis*. Taken together, isolate 1–4 was identified as a member of the *B. subtilis* group based on 16 S rDNA phylogeny and phenotypic traits.

### Antibacterial activity of AMPs derived from *B. subtilis* 1–4

**Fig. 2:**
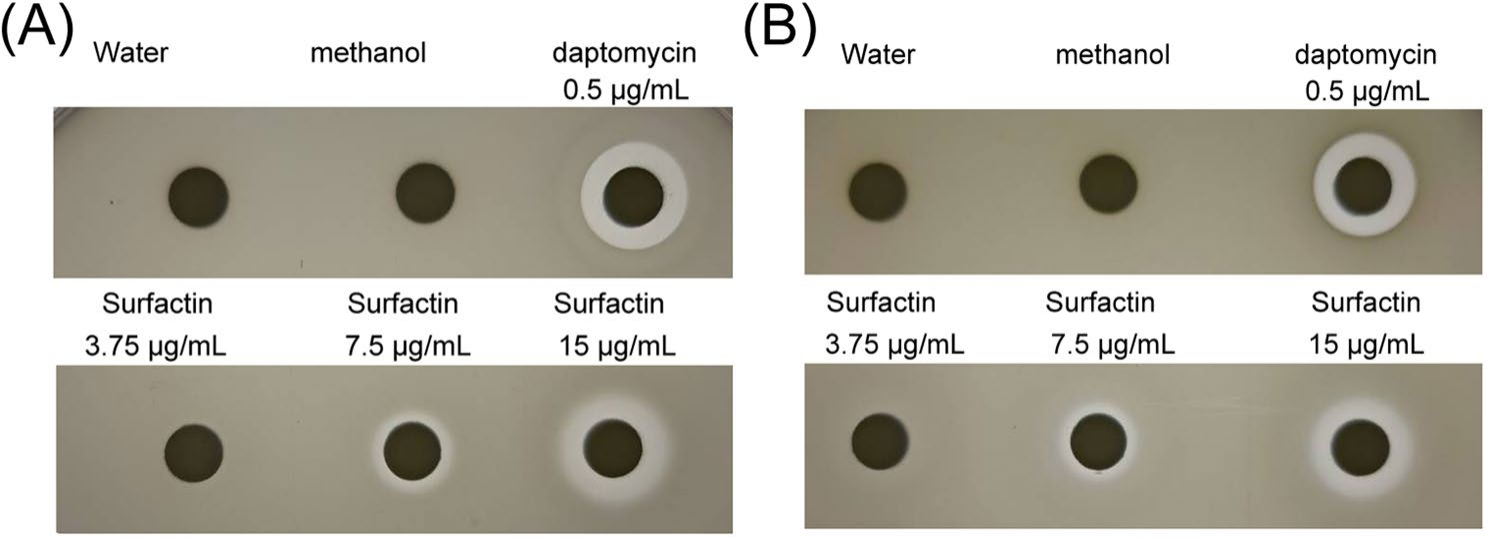
The antibacterial activity of surfactin from *B. subtilis* isolates 1–4 against (**a**) *S. intermedius* and (**b**) *S. aureus*. The LA plates with *S. intermedius* and *S. aureus* were incubated for 24 h at 37℃, respectively

**Fig. 3:**
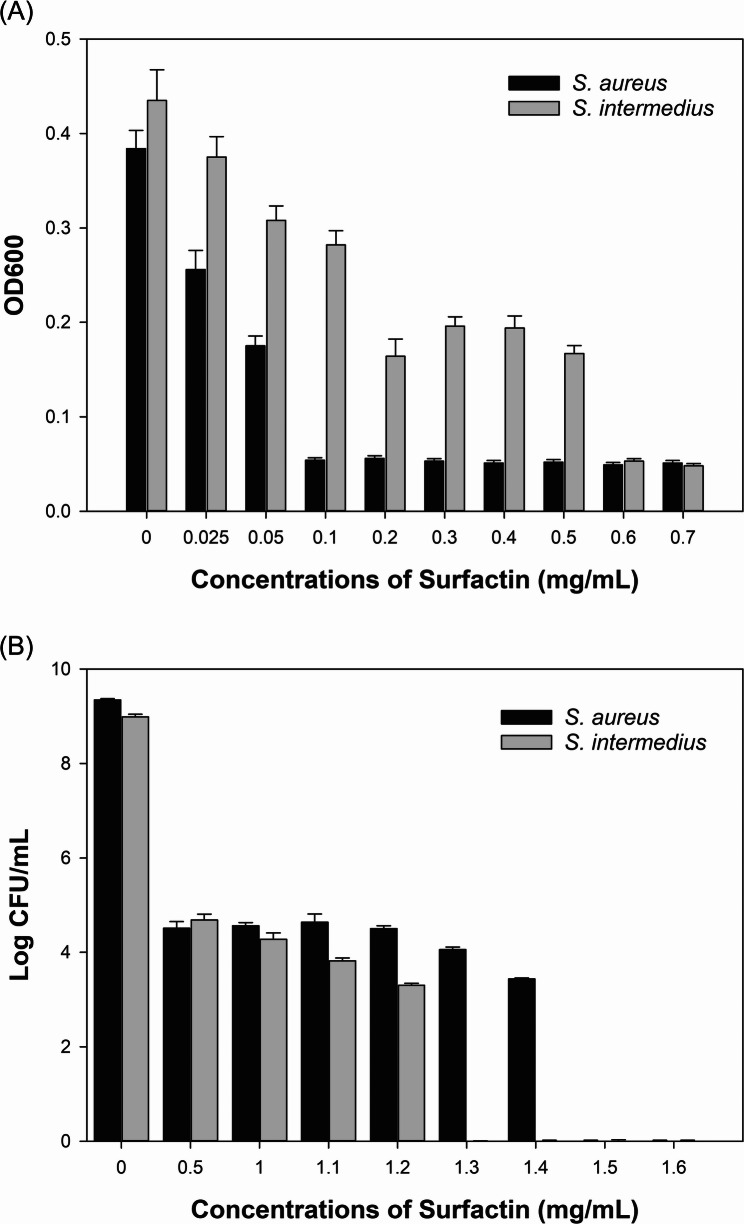
The minimum inhibitory concentrations (**A**) and minimum bactericidal concentrations (**B**) of surfactin from *Bacillus subtilis* 1–4 against *Staphylococcus aureus* (black bars) and *Staphylococcus intermedius* (grey bars). Data are presented as mean ± SD (*n* = 3). CFU, colony-forming units

AMPs extracted from the culture broth of *B. subtilis* 1–4 exhibited inhibitory activity against *S. aureus* and *S. intermedius*, as demonstrated by disc diffusion assays (Fig. 2). Clear inhibition zones were observed around discs loaded with AMPs, and the diameter of the zone increased in proportion to AMP concentration. In contrast, no inhibitory effects were observed for discs loaded with methanol or water controls. The MIC and MBC of AMPs derived from isolate 1–4 were determined to evaluate its antibacterial activity against *S. intermedius* and *S. aureus* and the results were given in Fig. 3. Against *S. intermedius*, the MIC and MBC values were 0.6 mg/mL and 1.3 mg/mL, respectively. In contrast, the MIC and MBC values against *S. aureus* were 0.1 mg/mL and 1.5 mg/mL, respectively.

### Identification of surfactin isomers in AMPs produced by isolate 1–4

**Fig. 4:**
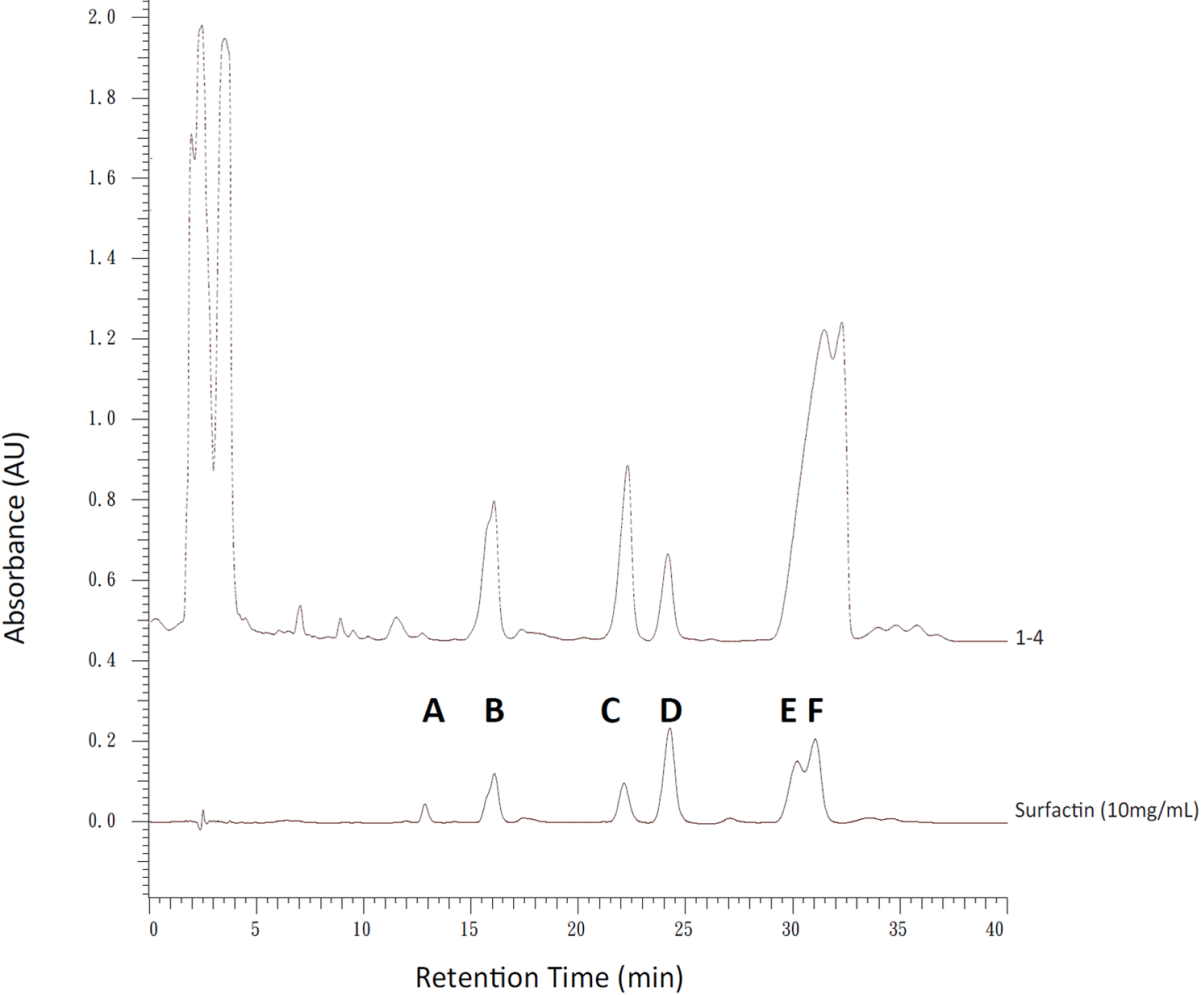
The elution profiles of AMP from 1–4 isolates of *B. subtilus* and the surfactin standard (10 mg/mL). The profiles were performed by HPLC with a C-18 reverse-phase column. Six isoforms of the surfactin were detected with retention times 12.8, 16, 22, 24, 29, and 30 min, and were labeled as **A** to **F**, respectively

AMPs precipitated from the culture broth of *B. subtilis* isolate 1–4 were dissolved in methanol, filtered, and analyzed by HPLC. As shown in Fig. 4, six distinct peaks were detected with retention times of 12.8, 16.0, 22.0, 24.0, 29.0, and 30.0 min. These retention times closely matched those of the six isomeric forms of a commercial surfactin standard, designated A through F, suggesting that the AMPs produced by isolate 1–4 comprise six surfactin isoforms.

### Bactericidal effect of surfactin on *S. intermedius* and *S. aureus*

**Fig. 5:**
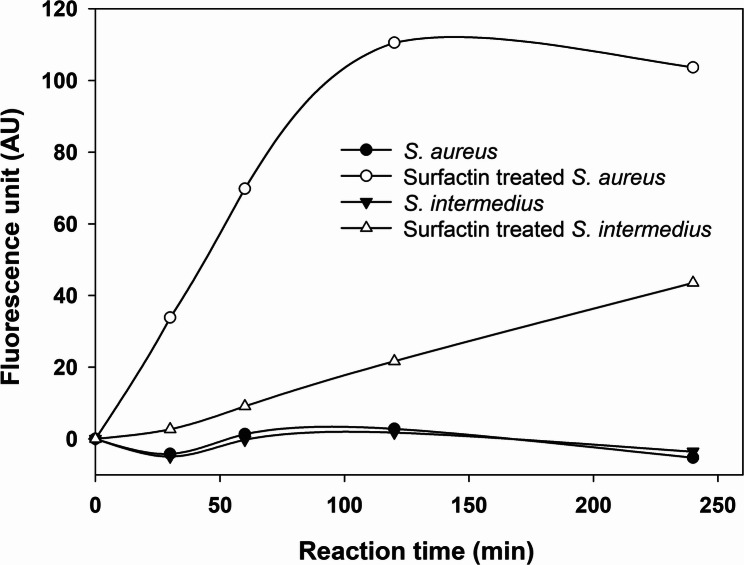
Cell membrane permeabilization of *Staphylococcus aureus* and *Staphylococcus intermedius* assessed by Sytox™ Green uptake. Bacterial membrane integrity was evaluated using a fluorometric assay at each species’ minimum bactericidal concentration (MBC) of surfactin. Fluorescence intensity was measured spectroscopically at 480 nm excitation and 522 nm emission wavelengths to detect Sytox™ Green binding, indicating compromised membrane permeability

The bactericidal activity of surfactin against *S. intermedius* and *S. aureus* was assessed by examining cell membrane integrity through the leakage of Sytox Green dye and the cytoplasmic proteins. The fluorescence intensity of Sytox Green in the supernatant of untreated *S. aureus* and *S. intermedius* remained unchanged over 4 h. When the concentration of surfactin was increased to MBC levels, the fluorescence intensity values in the supernatant of *S. aureus* and *S. intermedius* rose after 30 min of treatment (Fig. 5). For *S. intermedius*, the increase was statistically significant at both 30 min (*p* = 1.68 × 10^− 5^) and 60 min (*p* = 5.41 × 10^− 4^). The accumulation rate for *S. intermedius* was slower, whereas the accumulation rate for *S. aureus* was more pronounced, reaching its maximum after 2 h of treatment.

**Fig. 6:**
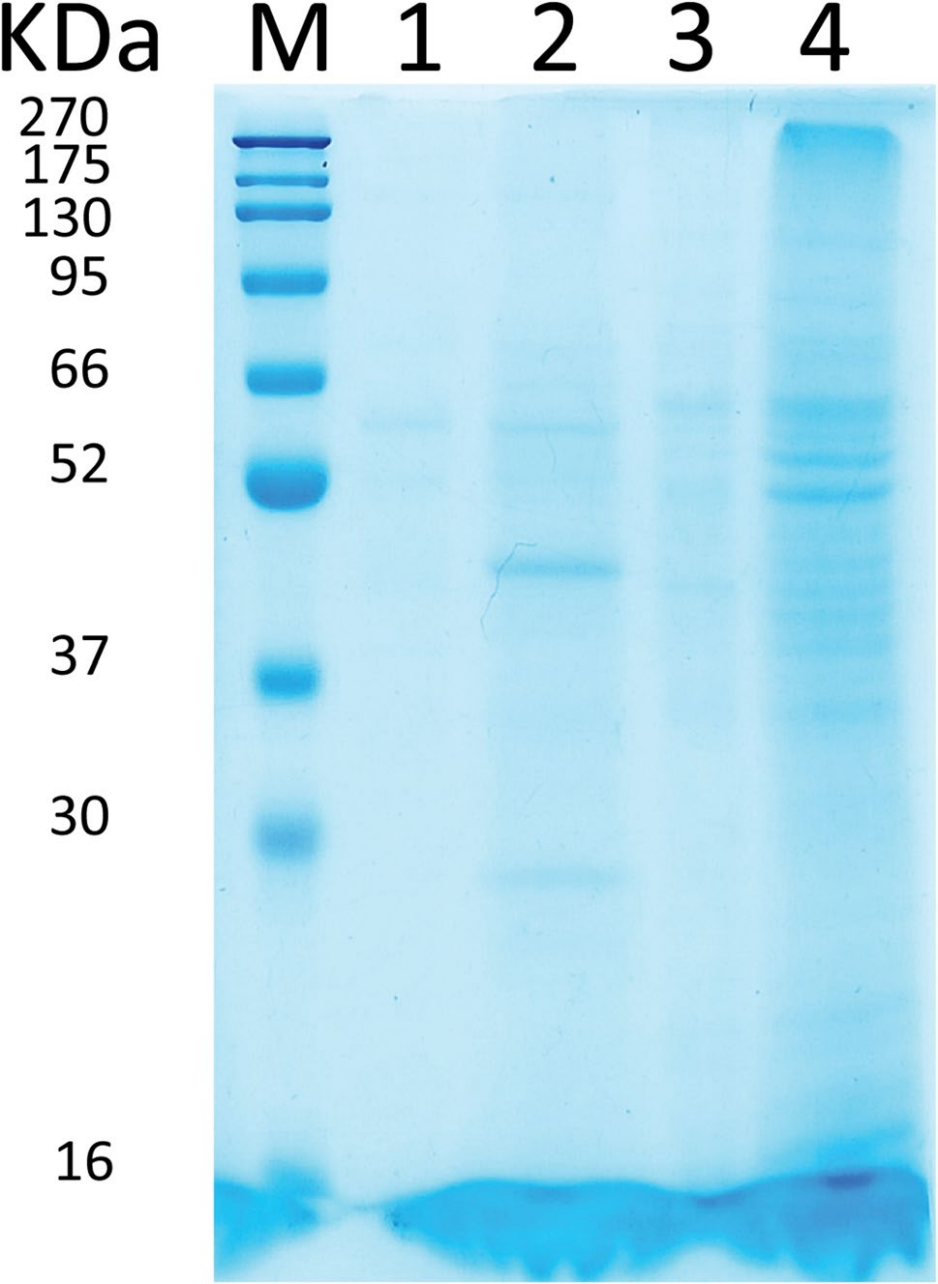
Tricine-SDS-PAGE analysis of endocytic protein release from *Staphylococcus aureus* and *Staphylococcus intermedius* following surfactin treatment. Representative gel image shows protein profiles from bacterial isolate 1–4 treated with surfactin at their respective minimum bactericidal concentrations and methanol controls. Lane M: molecular weight marker; Lane 1: *S. aureus* untreated control; Lane 2: *S. aureus* lysate after surfactin treatment; Lane 3: *S. intermedius* untreated control; Lane 4: *S. intermedius* lysate after surfactin treatment

The extracellular proteins of *S. aureus* and *S. intermedius* treated with the surfactin produced by isolate 1–4 at MBC levels were separated via SDS-PAGE. In the SDS-PAGE gel shown in Fig. 6, the bands of lanes 2 and 4 loaded with surfactin-treated mixtures were more numerous and significant than those of methanol-treated in both *S. aureus* and *S. intermedius* (lanes 1 and 3). Subsequently, several bands of surfactin-treated mixtures were randomly selected and identified by LC-MS/MS, and the results are provided in Table 1. Four cytosolic proteins, including phosphopyruvate hydratase and chaperonin GroEL, along with a membrane protein, L-glutamate gamma-semialdehyde dehydrogenase, found on the cytoplasmic side of the cell membrane, were detected in the extracellular fraction of *S. intermedius* cells treated with surfactin from isolate 1–4. Similarly, one cytosol protein was detected in the extracellular fraction of *S. aureus* treated with surfactin. Our findings indicate that the surfactin from isolate 1–4 damages the cell membrane, causing the release of proteins originally present in the cytoplasm and membrane, thereby exerting its bacteriolytic effect on the cells of *S. aureus* and *S. intermedius*.

**Table 1:** The identification of endocytic proteins released from *S. intermedius* and *S. aureus* cells using LC-MS/MS

Bacterium	GI Accession	Protein Name	Score*	Matched Peptide	Location
***S. intermedius***	517,998,076	L-glutamate gamma-semialdehyde dehydrogenase	64	GCTGAVVGYQPFGGFK	Cytoplasmic side of plasma membrane
	517,999,008	Dihydrolipoyl dehydrogenase	61	ILDSTGALNLQEVPK	Cytoplasm/cell membrane
	517,997,356	Chaperonin GroEL	60	EFTAPLITNDGVTIAK	Cytoplasm
	504,427,377	Phosphopyruvate hydratase	55	FEGTEDGVETILEAIK	Cytoplasm
	504,426,917	Type I glutamate ammonia ligase	36	GYTAVCNPLVNSYK	Cytoplasm
***S.aureus***	897,313,645	Trigger factor (ribosome-associated molecular chaperone; Tig)	48	TNLTLTAIAEAEK	Cytosol

*Mascot ion score is defined as − 10 × log10(P), where P is the probability of a random match. In Table 1, “Score” refers to the Mascot protein score; peptide-spectrum match significance was evaluated using the Mascot ion score (identity threshold). In our Mascot MS/MS searches, the identity threshold corresponding to *p* < 0.05 was an ion score > 36 for the *S. intermedius* dataset and > 37 for the *S. aureus* dataset. Only identifications meeting the identity threshold are reported in Table 1

### The impacts of surfactin on the diversity of gut microbial communities of pigeons

To investigate the impact of surfactin treatment on the changes in the gut bacterial community of pigeons, we analyzed the sequences of the full-length 16 S rDNA in pigeon feces collected in both the surfactin-treated group and the untreated control group. Samples were obtained at the start of the experiment (day 0; D0S for surfactin, D0CT for control) and after 7 days (day 7; D7S for surfactin, D7CT for control). Multiplex sequenced reads were deposited in the NCBI Sequence Read Archive (accession number PRJNA1290878).

**Fig. 7:**
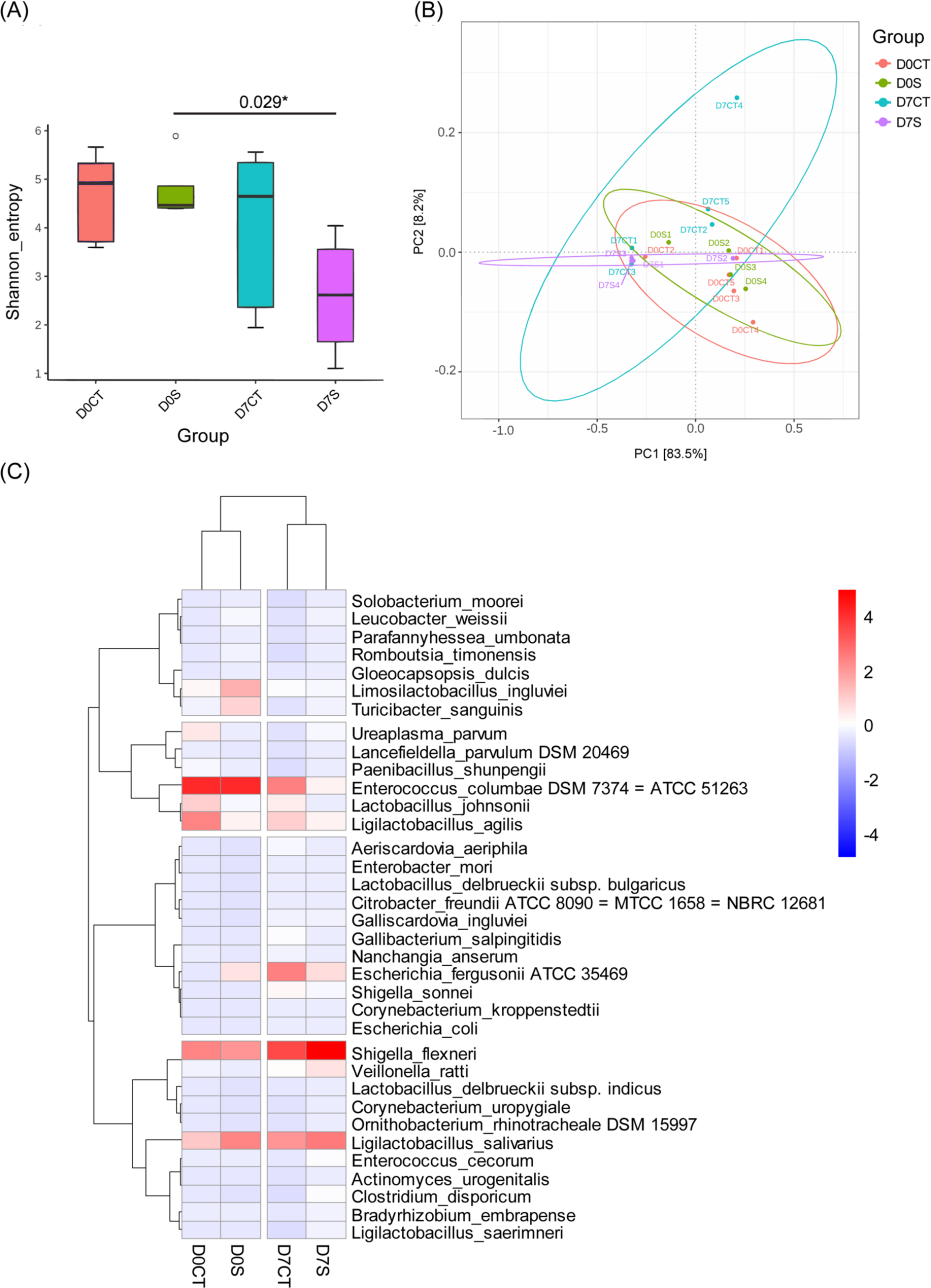
Gut microbiota diversity and composition in pigeons before and after surfactin treatment. (**A**) Alpha diversity (Shannon entropy) in four groups: D0CT (control, day 0), D0S (surfactin, day 0), D7CT (control, day 7), and D7S (surfactin, day 7). Significant difference is indicated (*p < 0.05). (**B**) Beta diversity assessed by principal coordinates analysis (PCoA) of weighted UniFrac distances, showing clustering patterns among the four groups. (**C**) Heatmap of the 35 most abundant bacterial species across all samples. Columns represent the four groups, and rows represent bacterial species clustered by similarity. The color scale denotes standardized relative abundance (z-scores), with red indicating higher and blue lower abundance

The alpha diversity estimated the diversity of the bacterial community in the guts within each sample (Fig. 7A). The alpha diversity significantly decreases in the Shannon index when comparing samples before and after surfactin treatment. However, no significant variations were detected between the control and surfactin treatment samples after 7-day treatment (*p* > 0.05).

The beta diversity was further analyzed to know the variation in the composition of bacterial communities among different sample groups (Fig. 7B). The PCoA based on UniFrac distances showed that the bacterial communities between D0S and D0CT groups, before treatment, had high similarities (ANOSIM, *p* = 0.962). After 7 days, the bacterial community of samples within the control group (D7CT) exhibited high diversity, whereas the surfactin-treated samples (D7S) showed a tendency towards increased similarity within the group during the same period. No significant variations were detected after 7 days of surfactin treatment (*p* = 0.195), while a significant variation (*p* = 0.015) was detected in the 7-day control group before and after 7 days using ANOSIM analysis. No significant difference was detected between D7CT and D7S at Day 7 (ANOSIM, *p* = 0.357).

At the species level, we focused on the top 35 most abundant bacterial species in each experimental group. Their relative abundances were visualized and compared among the 4 groups using a heatmap (Fig. 7C). There are 4 clusters of the top 35 bacterial species based on Pearson correlation. Notably, *Enterococcus columbae*, belonging to *Enterococcaceae*, had a higher abundance at the beginning of the experiment, with its lowest abundance in the D7S group. *Escherichia fergusonii*, belonging to *Enterobacteriaceae*, had the highest abundance in the control group after 7 days (D7CT); however, the increase was not significant in the surfactin treatment group after 7 days compared to the D0S group. *Shigella flexneri*, also belonging to *Enterococcaceae*, increased significantly after the 7-day experimental period and showed the highest abundance in the D7S group. Similarly, *Ligilactobacillus salivarius*, belonging to *Lactobacillaceae*, followed the same pattern. In contrast, *Lactobacillus johnsonii*,* Ligilactobacillus agilis*, and *Limosilactobacillus ingluviei*, also belonging to *Lactobacillaceae*, decreased after the 7-day experiment and had the lowest abundance in the D7S group.

## Discussion

Surfactin exhibited potent bactericidal activity primarily through disruption of membrane integrity, as evidenced by enhanced Sytox Green fluorescence and the release of intracellular proteins. This membrane-targeting mechanism aligns with surfactin’s amphipathic nature, facilitating its insertion into lipid bilayers and inducing membrane destabilization [35]. Unlike conventional antibiotics, surfactin does not act on metabolic pathways, but rather exerts rapid, physical damage to the cell envelope, reducing selective pressure for resistance development [36, 37]. The observed variations in bactericidal efficacy—reflected in differential MIC/MBC values and fluorescence kinetics between *S. aureus* and *S. intermedius*—suggest that membrane composition and associated factors such as surface charge and efflux activity modulate susceptibility (Figs. 5 and 6). Notably, *S. intermedius* exhibited slower fluorescence accumulation, potentially reflecting greater membrane resilience or structural differences.

Reported surfactin potency against staphylococci varies widely across studies; for example, MIC values of 0.512–1.024 mg/mL have been reported for *S. aureus*/MRSA, with MBC values typically 1 to 2 times of the MIC [38], whereas other assays report inhibitory activity at lower (20–100 of µg/mL) MIC concentrations [39, 40]. These published ranges encompass the MIC/MBC values observed here for *S. intermedius* (0.6/1.3 mg/mL) and *S. aureus* (0.1/1.5 mg/mL).

A finding of the β-diversity analysis was the significant difference between Day 0 and Day 7 shift observed within the control pigeons (ANOSIM, *p* = 0.015), despite high baseline similarity between groups before treatment (D0S vs. D0CT, *p* = 0.962) and no significant separation between the two groups at Day 7 (D7S vs. D7CT, *p* = 0.357). This pattern indicates substantial short-term, time-related variation in gut community structure that is biologically plausible in birds, as avian gut microbiota are sensitive to changes in captive environment, diet, feeding patterns, and stress physiology [41–43]. Indeed, brief captivity (on the order of days) has been reported to alter gut microbiome richness and composition in wild-caught songbirds. In addition, because ANOSIM can be sensitive to heterogeneity in within-group dispersion, the visually higher within-group variability observed in the Day 7 control samples could also contribute to the detected significance.

After accounting for the above time-related variability in controls, our data do not support a definitive causal conclusion that surfactin altered overall gut community structure over one week. Rather, the absence of a significant within-group β-diversity shift in the surfactin-treated pigeons (Day 0 vs. Day 7; *p* = 0.195), together with the lack of a significant between-group difference at Day 7 (*p* = 0.357), suggests that 7-day surfactin administration did not produce an additional detectable disruption of overall α- and β-diversity beyond background short-term variability. Although the PCoA visualization suggested a tendency toward greater within-group similarity in the surfactin-treated samples compared with controls, this pattern should be interpreted as a hypothesis-generating observation from a pilot study and requires confirmation using larger longitudinal cohorts and dose–response designs.

From a practical standpoint, the dietary inclusion level used in this study (1 g/kg feed) is within the range of surfactin doses explored in animal studies, although it is higher than some poultry feed-additive evaluations (e.g., 0.01% of the diet in broilers under necrotic enteritis challenge) [44]. In mammalian models, oral surfactin has commonly been administered at tens of mg/kg body weight and has been linked to improved intestinal barrier function and microbiota modulation in inflammatory and metabolic disease settings [45, 46]. Taken together, published evidence supports the biological plausibility that orally delivered surfactin can influence host–microbiota interactions without necessarily producing a broad collapse in community diversity; in this pigeon pilot, our results are most consistent with the absence of additional diversity-level disruption beyond short-term background variability. Future pigeon-specific work should therefore prioritize dose optimization (including lower inclusion levels), longer follow-up, and performance/safety endpoints to define a practical administration window for field use.

Although 7-day surfactin administration did not significantly alter α- or β-diversity, taxonomic shifts were observed. Enterococcaceae—particularly *E. columbae*—decreased, consistent with suppression of gram-positive taxa (Figs. 7A). While typically commensal, *E. columbae* can be opportunistic under stress or immunocompromise [47]; thus, reduced abundance may be relevant in susceptible hosts [48]. In contrast, *Shigella flexneri*, a gram-negative pathogen, increased in surfactin-treated samples (Fig. 7C), potentially reflecting indirect effects such as reduced *Lactobacillus* antagonism [49]. Although these trends were not statistically significant, they highlight testable hypotheses for future studies.

Beta diversity analysis revealed greater microbial homogeneity in surfactin-treated pigeons relative to controls (Fig. 7B), indicating that surfactin-treated pigeons did not show a greater degree of community disruption than that observed in untreated controls. For contextual comparison, broad-spectrum antibiotics are frequently associated with reduced microbial diversity and persistent perturbations across hosts (chickens, mice, horses, humans) [50–53]. Such antibiotic-induced dysbiosis can promote pro-inflammatory taxa, impair mucosal barrier function, and increase disease risk, with downstream metabolic and immune consequences that are often only partially reversible [54–56].

In avian hosts, antibiotics impair gut microbial metabolic function, notably reducing short-chain fatty acid synthesis (e.g., butyrate), which is essential for mucosal integrity and whose depletion further exacerbates dysbiosis [57, 58]. Moreover, early-life antibiotic exposure in chicks induced persistent reductions in microbial diversity and compromised developmental trajectories [59, 60]. Given that wild pigeons harbor zoonotic and multidrug-resistant pathogens [2], surfactin’s potential to modulate microbial communities without causing large-scale dysbiosis merits further investigation as an alternative to traditional antimicrobials.

## Conclusions

This study demonstrates that surfactin produced by *Bacillus subtilis* 1–4 compromises the cellular integrity of *Staphylococcus intermedius in vitro*. In pigeons, short-term surfactin administration did not result in additional detectable disruption of overall gut microbiota diversity or community structure beyond background temporal variability observed in controls.

Together, these findings extend current knowledge of surfactin as an anti-staphylococcal lipopeptide and provide a preliminary framework for exploring antibiotic-sparing strategies in avian hosts. However, confirmation in larger, well-controlled in vivo studies with longer follow-up and optimized dosing is required before definitive conclusions regarding microbiota modulation or practical application can be drawn.

## Supplementary Information

Supplementary Material 1.

## Data Availability

All data underlying this article are available in the article and in its online supplementary material. Additional data can be provided upon request. Sequences are deposited on SRA with BioProject ID: [PRJNA1290878](https://www.ncbi.nlm.nih.gov/bioproject/?term=PRJNA1290878).

## Abbreviations

AMPs: Antimicrobial peptides
ESI-Q-TOF: Electrospray ionization quadrupole time-of-flight
HPCL: High-performance liquid chromatograph
LEfSe: The Linear Discriminant Analysis Effect Size analysis
MBC: Minimum bactericidal concentration
MIC: Minimum inhibitory concentration
PCoA: Principal Coordinates Analysis
SDS–PAGE: Sodium dodecyl sulfate–polyacrylamide gel electrophoresis
