# Supplementary material for: Surfactin from Bacillus subtilis 1–4 disrupts the cell membrane of zoonotic Staphylococcus intermedius in vitro and maintains gut microbiota community in urban pigeons
Source: Gut Pathog. 2026 Apr 1;18:60. doi: 10.1186/s13099-026-00830-8 (PMC13397749; doi:10.1186/s13099-026-00830-8)
Supplement: Supplementary file 1 — Supplementary Material 1. [file 13099_2026_830_MOESM1_ESM.docx]

Supplementary File


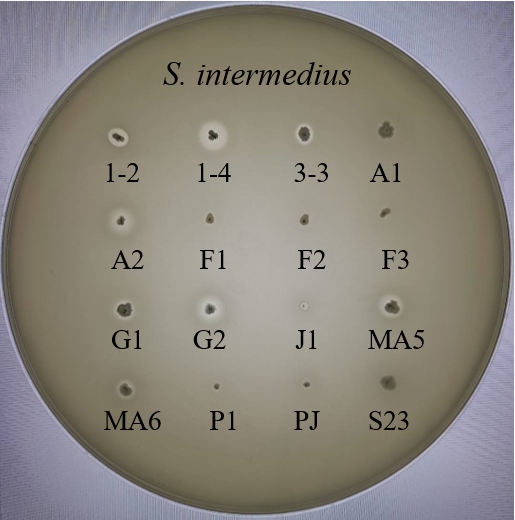

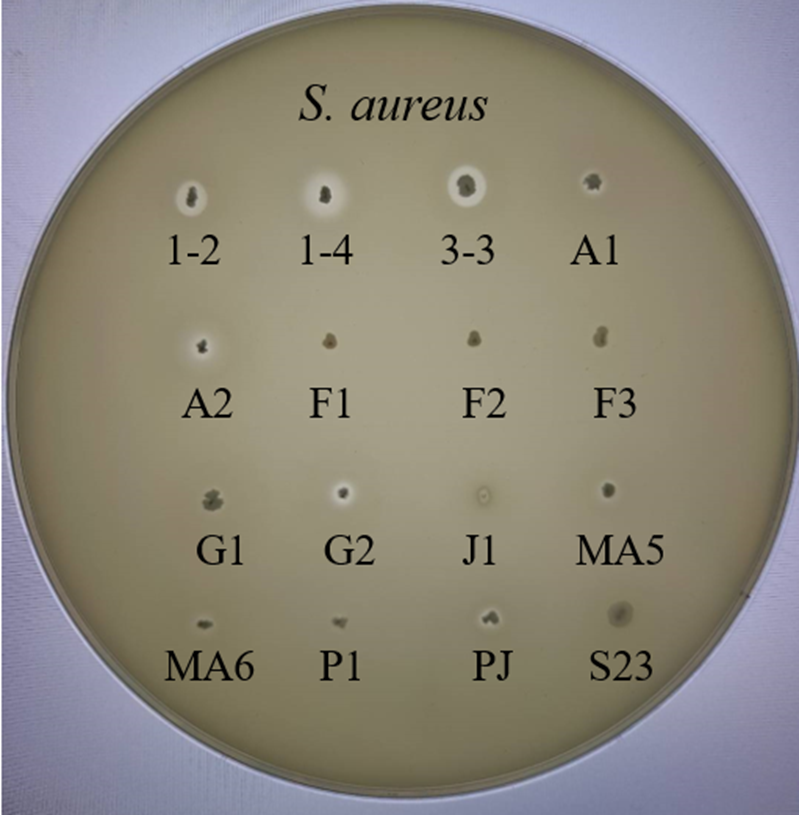
Figure S1

Figure S1. Screening of 16 bacterial isolates for antibacterial activity against pathogenic *Staphylococcus* *aureus* (A) and *S.* *intermedius* (B). Each isolate was inoculated onto agar plates containing either *S. aureus* (A) or *S. intermedius* (B). The diameters and clarity of inhibition zones were used as criteria for antimicrobial activity. Based on the results, isolate 1-4 exhibiting the strongest inhibitory effects was selected for further analysis.

Figure S2


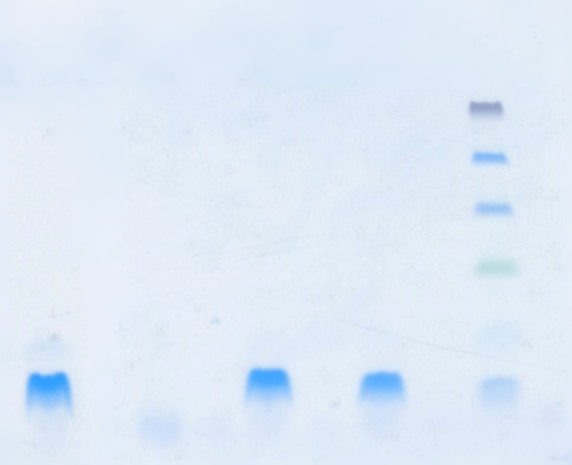


**1-4**

**40 kDa**

**25 kDa**

**15 kDa**

**10 kDa**

**4.6 kDa**

**1.7 kDa**

**M**

Figure S2. Tricine SDS-PAGE analysis of antimicrobial lipopeptides produced by isolates 1-4. The antimicrobial peptides extracts were separated on a Tricine SDS-PAGE gel and stained with Coomassie Brilliant Blue. Distinct bands were observed below 5 kDa, consistent with the molecular size of surfactin. Lane M: protein molecular weight marker.
